# Sequence-based prediction of protein binding regions and drug–target interactions

**DOI:** 10.1186/s13321-022-00584-w

**Published:** 2022-02-08

**Authors:** Ingoo Lee, Hojung Nam

**Affiliations:** School of Electrical Engineering and Computer Science, Gwangju Institute of Science and Technology, 123 Cheomdangwagi-ro, Buk-ku, Gwangju, 61005 Republic of Korea

**Keywords:** Drug–target interaction, Hit identification, Deep learning, Binding information, Protein sequence, Transformer

## Abstract

Identifying drug–target interactions (DTIs) is important for drug discovery. However, searching all drug–target spaces poses a major bottleneck. Therefore, recently many deep learning models have been proposed to address this problem. However, the developers of these deep learning models have neglected interpretability in model construction, which is closely related to a model’s performance. We hypothesized that training a model to predict important regions on a protein sequence would increase DTI prediction performance and provide a more interpretable model. Consequently, we constructed a deep learning model, named Highlights on Target Sequences (HoTS), which predicts binding regions (BRs) between a protein sequence and a drug ligand, as well as DTIs between them. To train the model, we collected complexes of protein–ligand interactions and protein sequences of binding sites and pretrained the model to predict BRs for a given protein sequence–ligand pair via object detection employing transformers. After pretraining the BR prediction, we trained the model to predict DTIs from a compound token designed to assign attention to BRs. We confirmed that training the BRs prediction model indeed improved the DTI prediction performance. The proposed HoTS model showed good performance in BR prediction on independent test datasets even though it does not use 3D structure information in its prediction. Furthermore, the HoTS model achieved the best performance in DTI prediction on test datasets. Additional analysis confirmed the appropriate attention for BRs and the importance of transformers in BR and DTI prediction. The source code is available on GitHub (https://github.com/GIST-CSBL/HoTS).

**Supplementary Information:**

The online version contains supplementary material available at 10.1186/s13321-022-00584-w.

## Introduction

Identifying drug–target interactions (DTIs) is a crucial step in drug discovery. As it is not feasible to test all chemical compounds against a given target protein, in silico prediction of possible active compounds using massive chemical libraries can increase the efficiency of drug discovery [1]. Thanks to the vast amount of information on drug compounds and their targets [2], as well as advances in computing power, researchers have been able to develop DTI prediction models using the proteochemometric (PCM) approach [3]. The PCM approach uses compound and protein descriptors, such as extended-connected fingerprints [4], CDK descriptors [5, 6], protein fingerprints [7, 8], and physicochemical properties for DTI [9], as the input features of machine learning models. The approach gives interpretable prediction results based on descriptors whose importance can be quantitatively calibrated to predict DTIs by a model [10–12]. Moreover, well-designed PCM models can give reliable DTI prediction results by modeling physicochemical interactions [13, 14].

As protein feature engineering for DTI prediction, identification of binding pockets/sites is important for prediction performance and comprehensive modeling [13–15]. Consequently, many computational models have been developed to identify binding pockets/sites. For example, the P2Rank algorithm [16] calculates the solvent-accessible surface of protein residues from the 3D structures of the Protein Data Bank (PDB) and builds features based on the calculated solvent-accessible surface and neighboring residues, which are used to rank protein residues as possible binding sites. DeepCSeqSite adopted convolutional neural networks (CNNs) to construct the prediction model on protein sequences that can recognize conserved motifs at binding sites, and this can be used to predict binding sites [17].

In addition to the DeepCSeqSite model, many CNN-based models are used to predict DTIs. DeepDTA was the first CNN-based model developed to predict binding affinity for kinase proteins using protein sequences and simplified molecular-input line-entry system (SMILES) notations for drug compounds [18]. Subsequently, the developers of DeepConv-DTI [19] showed that the latent representations of subsequences can be used as features to predict DTIs. Deep learning models outperformed previous machine learning-based models [3, 9, 20], while they learn sequential motifs by the model itself. However, deep learning can be a double-edged sword that gives accurate but uninterpretable predictions owing to its large number of parameters. Moreover, interdependency in protein sequence was not considered, meaning that the models predict DTIs based solely on motifs without a global context of proteins [21].

Recently, many attention-based models featuring protein sequences based on the local and global patterns of residues have been studied to remedy the aforementioned limitations. In particular, transformers utilize attention to assign relative importance to the residues of a protein sequence [22]. For DTI prediction, TransformerCPI [23] employs an encoder–decoder scheme to predict binding affinity. To model interdependency, MolTrans [24] leverages transformers built on frequent consecutive protein subsequences and SMILES notations to construct interaction maps for DTI pairs. DISAE [25] utilizes evolutionarily distilled sequence representations as inputs to ALBERT [26]. Some attention-based models train physical interactions between the substructures of ligands and binding sites of proteins to give better performance and interpretability. For example, DeepRelations [27] regularizes the attention of hierarchical recurrent neural networks to follow physical contacts, and MONN [28] builds a model to predict non-covalent interaction profiles and binding affinity. However, it does not consider long-range interaction between protein sequences, such as by using a hierarchical recurrent neural network and 1D-CNN for protein sequence embedding, and performance validation on external test datasets was not performed.

To address the above limitations, we propose a model to predict DTIs along what we refer to as binding regions (BRs), which are subsequences that include binding sites important to DTIs. The model, which we call Highlights on Target Sequences (HoTS), provides interpretable predictions of BRs and DTIs. To predict compound–protein BRs in HoTS, CNNs are employed to capture sequential motifs, and transformers are utilized to model interactions between sequential motifs and compounds. Thus, HoTS leverages the advantages of both CNNs and sequential models.

**Fig. 1:**
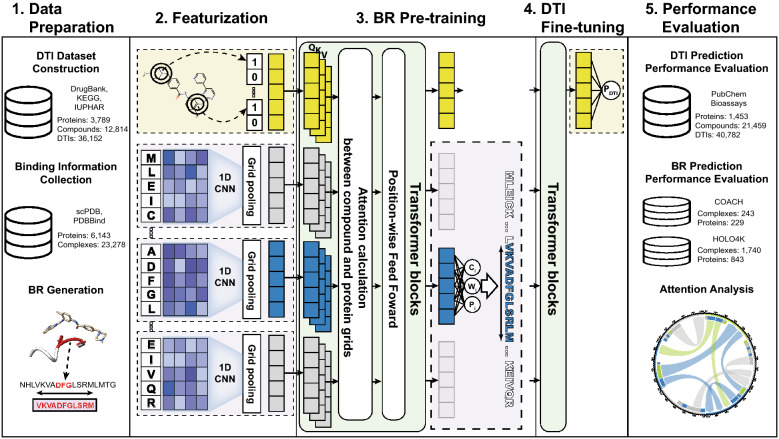
HoTS model overview. We first constructed a DTI dataset from DrugBank, KEGG, and IUPHAR. We also collected 3D complexes and their binding information (BI) to construct a BR dataset from scPDB and PDBBind. From the collected BI, we generated true BRs to train the BR prediction model. HoTS considers protein sequences of individual proteins and Morgan/circular fingerprints of drug compounds. Therefore, subsequences are extracted by a CNN, and the maximum values are pooled from each protein grid. Compound and protein grids are taken into transformers as queries, keys, and values to model interactions between subsequences and individual compounds. Closely related subsequences and compounds will have high attention, and as much as their attention, values of related subsequences/compounds are merged into new values. After passing the transformers, a compound token is used to predict DTIs, and individual protein grids are used to reflect the BRs. For DTI prediction, HoTS calculates a prediction score P_DTI_ ranging from 0 to 1, as well as center (C), length (W), and confidence (P) scores for BRs. We evaluated the DTI prediction performance using the PubChem Bioassay and BR prediction performance with the COACH and HOLO4K datasets

HoTS consists of two prediction models—one for predicting BRs and the other for predicting DTIs. To pre-train the BR prediction model, binding pockets/sites depicted in 3D complex databases are mapped to protein sequences and converted to BRs. A 1D-CNN with various window sizes on protein sequences then extracts motifs important to DTIs. These protein sequences are split into blocks of equal length, called protein grids, and the maximum convolution results are pooled from each grid. As sequential motifs are extracted, a compound token is generated from Morgan/circular fingerprints [4] and concatenated with the protein grids to reflect interactions between the compound and sequential motifs. Next, to model the dependencies between motifs and a compound, HoTS employs transformers on pooled CNNs and compound token results. For BR prediction, for each protein grid, the BR prediction model is pretrained to predict the location, size, and confidence score of the BRs. For DTI prediction, after passing additional transformers in the DTI prediction model, the compound token is fine-tuned to predict DTIs. We evaluated the DTI and BR prediction performance using independent test datasets and analyzed the attention of the transformers to understand the prediction mechanism. The overall flow of the model is illustrated in Fig. 1.

## Methods

### BR dataset construction

**Fig. 2:**
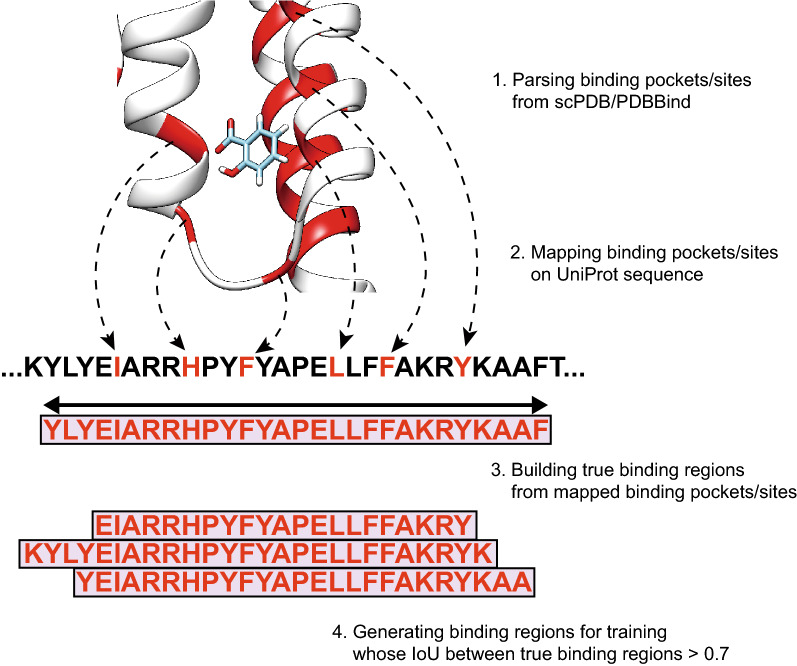
Generation of BRs from 3D complex databases. BRs were generated using by the following procedure. We collected BI from the scPDB and PDBBind databases. The collected BI was mapped onto the corresponding UniProt sequence, and as shown, it can be in non-consecutive sequences. To build consecutive subsequences including BI, we expanded the mapped BI to a length of 9 and merged the overlapped BI. In the BR training steps, we fed randomly generated BRs with an IoU larger than 0.7 to the prediction model

The concept of a binding pocket/site is ambiguous. Therefore, the definitions of binding sites and binding pockets [collectively referred to as binding information (BI)] vary by studies and databases. Usually, BI is defined as protein residues whose distance towards ligands is below a certain threshold. However, the thresholds for BI or distance measurements have been defined differently (e.g., number of atoms, mass center of ligand) in different studies [16, 29, 30]. In addition, motifs are not fully recognized as information for the distance towards ligands. For example, by the folding of a protein sequence and the binding mode of a ligand, BI can be discontinuous. We propose the concept of “binding regions (BRs)” as subsequences that include BI. To generate BRs as described in Fig. 2, we expanded the residues in BI and merged them into subsequences, defining them as BRs. As shown in Additional file 1: Fig. S1 we confirmed that the median percentage of residues in BRs was approximately threefold the median percentage of residues in BI, and generally, residues in BI tended to be concentrated rather than dispersed over the whole sequence. We first collected 3D complexes and generated BRs from scPDB [29] and PDBBind [30], employed as the BR training dataset. We regarded 3D complexes as DTI pairs. Multi-chain 3D complexes were split into single chains with their own interacting ligands, and each split pair was considered a DTI pair. In addition, we randomly split the collected BR dataset into a 9:1 training:validation dataset ratio. As an independent test dataset for performance validation, we utilized the COACH and HOLO4K test datasets from previous studies [16, 31, 32]. We collected BI as-is from scPDB [29] and generated ground truth BRs. Any PDB complexes that appeared in both the BR training and test datasets were removed from BR test dataset to prevent data leakage. Moreover, to evaluate the BR prediction performance for unseen proteins, we built a partial test dataset with 3D complexes with unseen proteins. We also measured the sequence similarity between BR training proteins and unseen proteins in the BR test dataset, as shown in Additional file 1: Fig. S2. Because our method depends solely on the protein sequence and fingerprint, rather than the 3D structures of proteins and ligands, the similarity of test proteins to training proteins will affect the prediction performance. We calculated the similarities between training proteins and unseen test proteins using the normalized Smith–Waterman score:

$${\text{SW}}_{{{\text{norm}}}} \left( {{\text{p}},{\text{p}^{\prime}}} \right) = \frac{{{\text{SW}}\left( {{\text{p}}, {\text{p}^{\prime}}} \right)}}{{\sqrt {{\text{SW}}\left( {{\text{p}},{\text{p}}} \right)} \sqrt {{\text{SW}}\left( {{\text{p}^{\prime}},{\text{p}^{\prime}}} \right)} }}.$$

Maximum similarity histograms between the BR test dataset (COACH and HOLO4K) and training dataset are shown in Additional file 1: Fig. S2. The statistics of the collected BR datasets are summarized in Table 1.

**Table 1:** Statistics of collected BR datasets

	Dataset	No. of complexes	No. of proteins
BR training dataset	scPDB (v2017)	15,258	4673
	PDBBind (v2018)	11,137	2615
	Total	23,278	6143
BR test dataset	COACH (unseen protein–ligand complexes)	243	229
	COACH (unseen proteins)	138	138
	HOLO4K (unseen protein–ligand complexes)	1740	843
	HOLO4K (unseen proteins)	692	425

### DTI dataset construction

To construct the DTI training dataset for the DTI prediction model, we collected DTIs from three databases: DrugBank [33], Kyoto Encyclopedia of Genes and Genomes (KEGG) [34], and the International Union of Basic and Clinical Pharmacology (IUPHAR) [35].

To select the hyperparameters of the DTI prediction model, we built an external validation dataset of DTIs unseen in the training phase. We collected DTIs from the MATADOR database [36] as positive DTIs, and DTIs that appeared as training DTIs were excluded from the validation dataset. To build a credible negative dataset, we obtained negative DTIs using the method described by Liu et al. [37].

To evaluate the performance of the model in DTI prediction, we constructed independent test datasets from the PubChem BioAssay [38], which includes experimental results. To evaluate model performance, we collected two independent datasets from the PubChem BioAssay from a protein perspective. We regarded active compounds of ligand binding assays with a dissociation constant as positive DTIs, and the same number of inactive compounds with other types of assays were sampled as negative DTIs [19]. For the first dataset, we gathered all druggable proteins from DGIdb [39] to evaluate our general DTI prediction performance, which we called DTI test dataset 1. Second, as a subset of DTI test dataset 1, we gathered DTIs whose proteins were of the same SCOPe families [40] as proteins in the BR training dataset to show that BR training can increase DTI prediction performance for proteins whose structural motifs are trained; we called this DTI test dataset 2. The statistics of the collected DTI datasets are summarized in Table 2.

**Table 2:** Statistics of collected DTI datasets

	Dataset	No. of compounds	No. of proteins	No. of positive DTIs	No. of negative DTIs
DTI training dataset	DrugBank (v2020)	5080	2685	14,679	−
	KEGG (v2020)	4033	772	11,835	−
	IUPHAR (v2020)	6295	2017	14,282	−
	Total	12,814	3789	36,152	72,304
DTI validation dataset	MATADOR	252	145	307	−
	Liu et al	255	410	−	508
	Total	499	538	307	508
DTI test dataset	DTI test dataset 1^a^	21,459	1453	20,391	20,391
	DTI test dataset 2^b^	4991	134	5001	5001

^a^PubChem Bioassays whose druggable proteins were from DGIdb

^b^Subset of DTI test dataset 1, whose proteins have the same SCOPe family as the BR training dataset

### Proposed HoTS prediction model

For proteins, HoTS first extracts sequential motifs, as implemented in DeepConv-DTI [19]. Next, it splits the CNN results into grids and then pools the maximum values in each grid. The pooled maximum values are then fed into a fully connected layer. As a result, proteins are represented as sequences of grid encodings based on the convolution results, which are more suitable for predicting BRs and model interdependencies. The compounds are converted to Morgan/circular fingerprints [4] of 2048 bits with a radius of 2. Compound fingerprints are then passed through fully connected layers, forming what we call a compound token, and are attached to their respective protein grid encodings. Thus, the size of the concatenated feature representing compound and sequence will be H × (1 + ⌈protein_length/grid_size⌉), where H denotes the size of the hidden dimension. Positional encoding is added to this compound–grids feature to help the transformers comprehend the relative positional information. These compound–grids features are then taken as the inputs of the transformer blocks. By passing this information through the continuous transformer blocks, the compound token will reflect the overall interaction with the target protein, and the protein grid encodings will represent the interactions and selectivity to ligands. Thus, the prediction of BRs can be represented as

$${\text{c}}_{{\text{g}}} ,w_{g} , p_{g} = \sigma \left( {f_{{{\text{BR}}}} \left( {{\text{TransformerBlocks}}_{{{\text{BR}}}} \left( {h_{{{\text{grids}}}} } \right)} \right)} \right),$$

where $h_{{{\text{grids}}}}$ denotes the protein grids parts of the compound–grids feature, $f_{{{\text{BR}}}} \left( \cdot \right)$ denotes the dense layers for BR prediction, and ${\upsigma }\left( \cdot \right)$ denotes the sigmoid function. Predictions ${\text{c}}_{{\text{g}}} ,w_{g} , p_{g}$ are given for each protein grid; they represent center, width, and confidence score, respectively. Prediction of DTIs can be represented as

$${\text{P}}_{{{\text{DTI}}}} = \sigma \left( {f_{{{\text{DTI}}}} \left( {{\text{TransformerBlocks}}_{{{\text{DTI}}}} \left( {{\text{TansformerBlocks}}_{{{\text{BR}}}} \left( {h_{{{\text{compound}}}} } \right)} \right)} \right)} \right),$$

where $h_{{{\text{compound}}}}$ denotes the compound token that is a part of the compound–grids feature, and $f_{DTI} \left( \cdot \right)$ denotes the dense layers for DTI prediction. Finally, ${\text{P}}_{{{\text{DTI}}}}$ gives prediction score for DTI pair. A more detailed illustration of the model, transformers, and training scheme is shown in Additional file 1.

### Prediction model of binding regions

In the field of computer vision and image processing, object detection involves the detection and classification of individual objects from an image. Simple object detection models slice images into grids and predict B bounding boxes and confidence scores in each grid. For example, the You Only Look Once (YOLO) model, which is a single-shot detector, employs a simple CNN-based model to retrieve objects from images [41, 42]. The BR prediction model in HoTS relies on a similar architecture to detect BRs across three model constructs: CNN layers for identifying sequential motifs, transformers for modeling interdependency, and fully connected layers for the actual prediction of BRs. The BR prediction model predicts the center, width, and confidence scores of BRs, as well as a prediction score ranging from 0 to 1. These are then reconstructed to their original values. The centers of the BRs (Center_g_) are reconstructed by

$${\text{Center}}_{g} = s_{{\text{g}}} + {\text{size}}_{{{\text{grid}}}} \cdot c_{g} ,$$

where c_g_ is a prediction for the BR location, and s_g_ is a starting index of the grid. As a result, Center_g_ represents the location of the predicted BR in the protein sequence.

The widths of the BRs (Width_ig_) are reconstructed by

$${\text{Width}}_{ig} = {\text{r}}_{{\text{i}}} e^{{w_{g} }} ,$$

where w_g_ is a prediction for the BR width, and r_i_ is the predefined BR width. e is Euler’s number. Therefore, Width_ig_ represents the width of the predicted BR.

In image classification models using region of interest pooling methods, such as Faster R-CNN, both objects and non-objects are sampled and passed through the classification model, leading to class imbalance (i.e., a small number of objects in one class and a much larger number in the other) [43]. Meanwhile, single-shot detectors, such as RetinaNet and YOLO, use the focal loss to dynamically control weights for object classification and prevent class imbalance [44]. Similarly, as shown in Additional file 1: Fig. S1, the percentages of amino acids at binding sites and BRs are small, which would induce a class imbalance in the BR prediction model and result in a decrease in the prediction performance. Accordingly, we utilize focal loss to address the class imbalance in BR detection, defined as follows:

$${\text{FL}}\left( {{\text{p}}_{{\text{t}}} } \right) = { } - \left( {1 - {\text{p}}_{{\text{t}}} } \right)^{\gamma } \log \left( {p_{t} } \right),\; {\text{where}}\; {\text{p}}_{{\text{t}}} = \left\{ {\begin{array}{*{20}ll} {p, \qquad \qquad if \,y = 1} \\ {\left( {1 - p} \right), \quad\,\,\, elif \,y = 0} \\ \end{array} } \right.,$$

where the weights of loss are controlled dynamically to reduce the class imbalance. The losses for the centers and widths of BRs are calculated using the mean absolute error. Therefore, the total loss for BR prediction is calculated as

$${\text{L}}\left( {{\text{c}},{\text{ w}},{\text{ p}}} \right) = \lambda_{reg} \left( {\left| {c_{true} - c_{pred} } \right|_{1} + \left| {w_{true} - w_{pred} } \right|_{1} } \right) + \lambda_{conf} {\text{FL}}\left( {p_{t} } \right),$$

where | |_1_ represents L1 loss, and λ_reg_ and λ_conf_ are weights for the regression and focal loss, respectively.

### Prediction of drug–target interactions

By passing through transformers to model compound–protein interactions, compound tokens sum up protein grid encodings via attention for drug–protein interactions. In the DTI prediction model, we stack more transformers for compound tokens on the BR prediction model to better reflect the DTIs over BR prediction. With the aforementioned mechanism of attention on drug interactions, the transformers act as “aggregators” for DTI prediction. After passing all transformers in HoTS, compound encoding is used to predict DTIs across fully connected layers.

### Training scheme

We firstly pre-trained the BR prediction model, which consists of (1) a CNN layer to extract interacting motifs, (2) transformers to model interdependency, and (3) fully connected layers to predict BR. After BR prediction performance converges, we stacked additional transformers on the BR prediction model to build the DTI prediction model. Therefore, while former transformers are used to predict BR, later transformers aggregate interaction information on the compound tokens to predict DTI. Next, we fine-tuned the BR prediction model to predict DTIs with the DTI training dataset. During DTI fine-tuning, we alternatively trained the BR prediction and DTI prediction to reduce the discrepancy between the BR prediction model and the DTI prediction mode. Because the BR training dataset size is smaller than the DTI training dataset, we trained the BR prediction model three epochs for every one DTI prediction model epoch.

### Performance evaluation

#### Intersection over union

We utilized intersection over union (IoU) to evaluate BR prediction. IoU is the ratio of the overlapping region between the ground truth BR and predicted BR (intersection) to the combination of the ground truth BR and predicted BR (union). We regarded a predicted BR with an IoU larger than 0.5 as a true positive.

#### Average precision

To evaluate the BR prediction performance, we assessed the average precision (AP) of the model for object detection. AP was calculated over several steps. First, all predictions from the evaluation dataset were collected and sorted according to their confidence scores. Second, for every sorted prediction, precision and recall were calculated using IoU, as with the calculation of area under precision–recall (AUPR) curve. The AUPR curves were interpolated by the following formula:

$${\text{p}}_{{{\text{interp}}}} \left( r \right) = \mathop {\max }\limits_{{\tilde{r} \ge r}} p\left( {\tilde{r}} \right),$$

where p is precision and r is recall.

Third, the AP is calculated by averaging the precision. We can regard AP as an interpolated AUPR for object detection problems. In short, AP reflects how accurately the model detects BRs across an entire protein sequence.

#### Top-n and top-(n + 2) prediction

Although AP is an effective measurement of the performance of BR detection, it is an overall performance evaluation metric rather than an actual prediction for the BR of a ligand. Therefore, in the prediction step, we adopt top-n and top-(n + 2) prediction from P2Rank [16]. Because we predict BRs, which are located separately in a sequence but indicate the same binding pocket, which are closely located in a 3D complex, we consider each BR prediction as a BI prediction. Therefore, as in previous methods, we take the BR predictions with the highest confidence scores as the number of interacting ligands to evaluate our model (top-n). For top-(n + 2) prediction, two additional predicted BRs are counted to determine whether BR prediction succeeds in predicting the true BR. We regarded the predicted BR as correct when it covered half of the true BR. Finally, we evaluated the success rate of BR prediction considering the top-n and top-(n + 2), where any true BRs are considered correct with top-n or top-(n + 2) BR predictions.

### Attention analysis

#### Statistical analysis for the attention of compound tokens for BRs/non-BRs

As mentioned above, protein grids, pooled convolution results on a protein sequence are taken as transformer inputs, and compound tokens are attached before the protein grid to predict DTIs. We assumed that compound tokens are more concentrated on BRs. To verify our hypothesis, we first collected the maximum attention values of the compound tokens from the transformers. We distinguished the maximum attention values of BR and non-BR for each ligand–protein complex. With the collected maximum values, we fitted two Gumbel distributions for the maximum BR/non-BR attention values. Finally, we conducted a Kolmogorov–Smirnov test between the BR/non-BR Gumbel distributions to verify the statistical differences between the attentions of the BR/non-BR compound tokens.

#### Circos plot of attention

For more detailed inspection, we visualized the attention values using a Circos plot [45, 46]. Compound tokens and protein grids were represented as sectors of a Circos plot. We colored sectors by their type, where the compound token is yellow, BR is blue, and non-BR is gray. Sectors were arranged clockwise from compound token to last protein grid; note that compound token and last protein grid are attached in the Circos plot but they are actually not adjacent in the transformer input. We take only the upper 90% of the attention values for better visualization, neglecting unnecessary attention values. The attention value from the query to the key is represented as a stroking line starting from the source sector to the target sector, whose color follows the source sector and width represents the relative attention value. A more detailed explanation of the Circos plot with an example is provided in Additional file 1: Fig. S3.

## Results and discussion

### Training results

We trained the HoTS model under the following scheme. First, we trained the BR prediction model using CNN layers and transformers. After the pre-training steps, we added more transformers to reflect the DTIs based on pre-trained information for BRs. In training for DTIs, the initial validation performance of the BR prediction model dropped, as shown in Additional file 1: Fig. S4. Therefore, we further trained the BR prediction model using additional DTI training epochs. The untrained BR prediction model showed a low AP for BRs. After further pre-training epochs, the AP values for the BR prediction model in the validation dataset gradually increased. However, as stated above, the AP dropped significantly at the first DTI training epoch, although AP values for additional DTI training epochs converged following the trend of those for the BR prediction epochs. Given the observed convergence in model performance, we interpret that the BR and DTI prediction models shared common features. Finally, we achieved an AP of 62.28% for the BR validation dataset, consisting of a randomly sampled 10% of the collected dataset. Using the selected hyperparameters summarized in Additional file 1: Table S1 the DTI prediction model exhibited an area under the receiver operating characteristic curve of 0.8542 and an AUPR of 0.8232 on the BR validation datasets.

### Prediction performance for binding regions

We evaluated performance on the COACH and HOLO4K datasets, where complexes and proteins, which are redundant to BR training dataset, were removed for the validity of the performance evaluation. Previous 3D information-based methods (Fpocket [47], SiteHound [48], MetaPocket 2.0 [49], DeepSite [50], P2Rank [16]) regard prediction as true positive when the distance between the center of the pocket and any ligand atom (DCCcriterion) is less than 4 Å. However, the proposed HoTS, only takes a protein sequence and predicts BRs. We gathered as many predictions as the number of ligands (top-*n* success rate, where *n* indicates the number of interacting ligand in complex) for comparison, following the previous evaluation methodology [16, 51, 52]. In addition, we considered two additional BR predictions for the top-(*n* + 2) success rate. With top-*n* or top-(*n* + 2) predictions, we regarded success as the prediction of BRs covering true BRs. As shown in Tables 3 and 4, HoTS showed better performance than the other methods on the COACH dataset. For the HOLO4K dataset, although it could not outperform 3D structure-based methods, HoTS achieved reasonable performance for BR prediction, outperforming some previous methods without 3D information of proteins. HoTS showed better performance on COACH than HOLO4K, while their percentages of BI/BR and sequence similarity to BR training proteins were similar. We speculate that the performance gap is due to the difference between dataset compositions. HOLO4K mainly contains larger multimers, while COACH contains single chains with smaller proteins [16], similarly to our BR training dataset.

**Table 3:** BR prediction performance on the COACH test dataset

Name	COACH (unseen protein–ligand complexes)		COACH (unseen proteins)	
	Top-*n* success rate (%)	Top-(*n* + 2) success rate (%)	Top-*n* success rate (%)	Top-(*n* + 2) success rate (%)
Fpocket	59.2	63.5	55.2	59.0
SiteHound	54.8	74.1	52.2	69.6
MetaPocket 2.0	68.3	80.9	64.1	76.3
DeepSite	61.8	67.8	57.5	64.9
P2Rank	74.7	82.0	73.1	79.9
HoTS (proposed)	66.3 ± 0.9	85.26 ± 0.8	59.9 ± 1.3	81.4 ± 0.9

**Table 4:** BR prediction performance on HOLO4K test dataset

Name	HOLO4K unseen protein–ligand complexes)		HOLO4K (unseen proteins)	
	Top-*n* success rate (%)	Top-(*n* + 2) success rate (%)	Top-*n* success rate (%)	Top-(*n* + 2) success rate (%)
Fpocket	61.1	67.2	58.4	62.6
SiteHound	64.9	78.2	57.9	70.3
MetaPocket 2.0	70.9	82.2	65.4	75.8
DeepSite	60.6	65.7	64.3	70.5
P2Rank	78.1	89.3	74.4	86.5
HoTS (proposed)	61.4 ± 0.7	79.1 ± 0.4	53.2 ± 0.6	71.7 ± 0.6

We also evaluated the prediction performances on HOLO4K with different similarity thresholds, as shown in Additional file 1: Table S2. As a result, for the COACH dataset, we showed that HoTS BR prediction performance is rigid to protein sequence similarity. For HOLO4K, although the BR prediction performance of HoTS decreased with similarity, HoTS achieved 66.53 ± 0.78% top-(n + 2) prediction performance with a 0.5 similarity threshold.

**Fig. 3:**
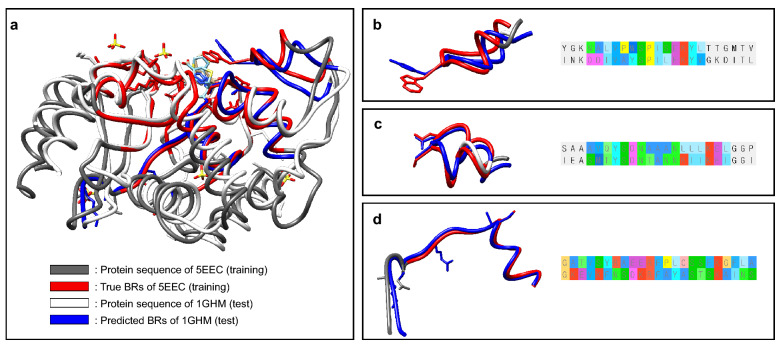
Prediction and visualization of binding regions on homologous 3D complexes with low sequence similarity. We visualized BR prediction for beta-lactamase in the test dataset (PDB ID: 1GHM, UniProt accession: P00807, white colored), which is homologous to the training protein (PDB ID: 5EEC, UniProt accession: Q9F663, dark gray colored) with low sequential identity/similarity. **a** Structural alignment results for 5EEC and 1GHM. True BRs of 5EEC are colored red, and the predicted top-3 BRs are colored blue for IGHM. **b**–**d** Structural and sequential alignment of predicted BRs, showing that HoTS can predict the same BRs with different residues

In addition to quantitative evaluation of performance, we analyzed the 3D complexes of the test dataset with low sequence similarity (0.4321) but high structural similarity. We compared beta-lactamases of different organisms: Klebsiella pneumonia in training (PDB ID: 5EEC [53], UniProt ID: Q9F663, white-colored), and Staphylococcus aureus in testing (PDB ID: 1GHM [54], UniProt ID: P00807, cyan colored). As shown in Fig. 3a, despite their low sequence similarity, the test structure is aligned on the training protein with a root mean square deviation of 1.177 Å by MatchMaker [55] of UCSF Chimera [56]. True BRs are colored red for 5EEC from the BR training dataset, and the top-3 predicted BRs are colored blue for 1GHM from the BR test dataset. We can see that the top-3 predicted BRs are located near the interacting ligand, covering the true BRs of 5EEC. In addition, as we can see with the aligned sequence with AliView [57] in Fig. 3b–d. HoTS predicted the same BRs, despite their differences in residues and ligands.

### Prediction performance for drug–target interactions

**Fig. 4:**
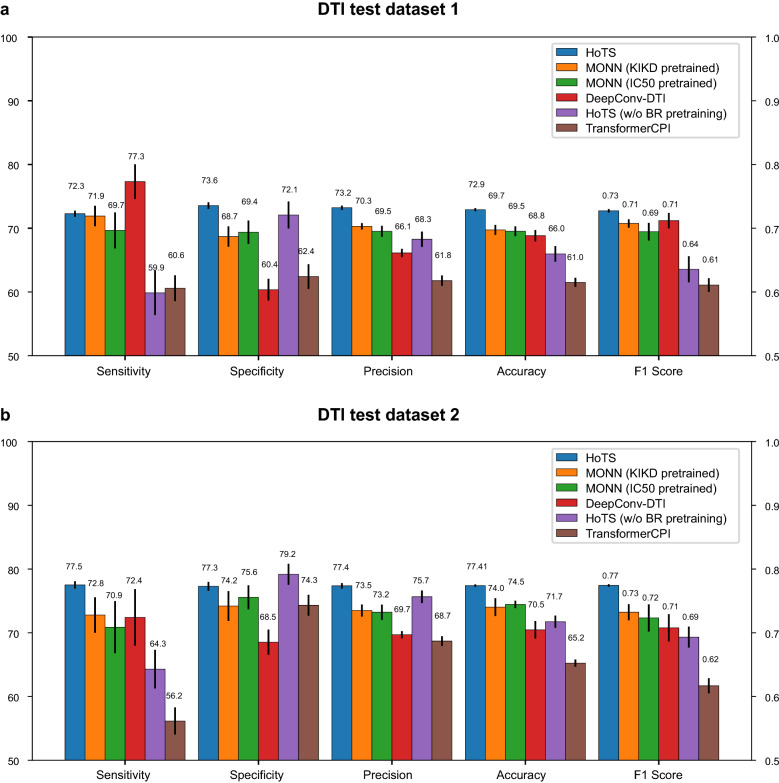
Prediction performance for drug–target interactions in the independent test datasets. DTIs were evaluated using PubChem Bioassays. Error bars stand for standard error. **a** DTI prediction performance for bioassays with all druggable proteins derived from DGIdb. HoTS overall outperformed the other models and HoTS without BR training, showing that BR training indeed improves DTI prediction performance. **b** DTI prediction performances for a subset of bioassays on DTI test dataset 1, whose proteins have the same SCOPe family as the BR training dataset. HoTS overall greatly outperformed the other models and HoTS without BR training, showing that BR training indeed helps DTI prediction with the same structural motifs

Next, we compared the performance of the HoTS model in predicting DTIs with those of MONN [28], DeepConv-DTI [19], TransformerCPI [23], and HoTS without BR training (model only). First, MONN was pretrained by the two types of binding affinities (KIKD and IC50) separately, with their non-covalent bonds between proteins and the ligands from PDBBind, resulting in two models, MONN (KIKD pretrained) and MONN (IC50 pretrained). We fine-tuned each pretrained MONN model to predict DTI using the DTI training data. Next, DeepConv-DTI and TransformerCPI are also trained with the DTI training dataset without BR pre-training. To evaluate the effect of BR pre-training to DTI prediction performance, we also trained the HoTS model without BR pre-training. The HoTS model with BR pre-training showed the highest performance on DTI test datasets 1 and 2 (Fig. 4), with much greater precision and specificity, suggesting that training BRs helps more precise predictions. Regarding the MONN model, the pretrained MONN models showed better performance than the other previous models, representing the importance of BI pre-training in DTI prediction. However, because MONN does not consider long-distance interactions in proteins and having high complexity of attention calculation for DTI prediction (the length of protein sequence by the number of atoms in ligand), we speculate that the problems above reduced DTI prediction performance [24, 25, 27]. The other transformer-based models (TransformerCPI and HoTS without BR training) showed no better performance than the simple convolution-based model (DeepConv-DTI). DeepConv-DTI utilizes CNN layers to extract important sequential motifs of protein sequences, and their maximum values are utilized as protein representations. However, DeepConv-DTI does not model interdependency or long-distance interactions [21], and it does not train important motifs explicitly. HoTS overcomes these limitations by employing transformers that model interactions between compounds and protein grids and explicitly training BRs. However, the mere use of transformers does not guarantee a performance increase. For example, although TransformerCPI utilizes 3-mers of protein sequence data as protein features, researchers have shown that full-length representations of a protein sequence can lower DTI prediction performance [24, 25]. In contrast, HoTS extracts interacting motifs and pools maximum convolution results in protein grids to reflect sequential motifs important to DTIs while reducing the complexity of attention calculations.

On DTI test dataset 1, the HoTS model trained with BRs also exhibited better DTI prediction performance on general druggable proteins from DGIdb [39] (Fig. 4a), indicating that training with BRs helps in DTI prediction in general. On DTI test dataset 2, for proteins of the same SCOPe family [40], the HoTS model trained with BRs showed significantly better accuracy and F1 scores (Fig. 4b). Training BRs worked for both exact motifs and sequentially/structurally/functionally similar motifs, reflecting the generalization of the trained BRs. Because binding motifs are evolutionarily conserved, the DTI prediction model, which employs BRs trained in the BR prediction model, is able to concentrate on learned BRs, showing great performance improvements over other available DTI prediction models.

We also trained and tested our model on previous benchmark datasets, BindingDB dataset [58], Human dataset, and *Caenorhabditis elegans* dataset [59]. Performance on external benchmark datasets are summarized in Additional file 1: Tables S3–S5. As reported, the proposed model mostly outperforms previous models.

### Analysis of attention

**Fig. 5:**
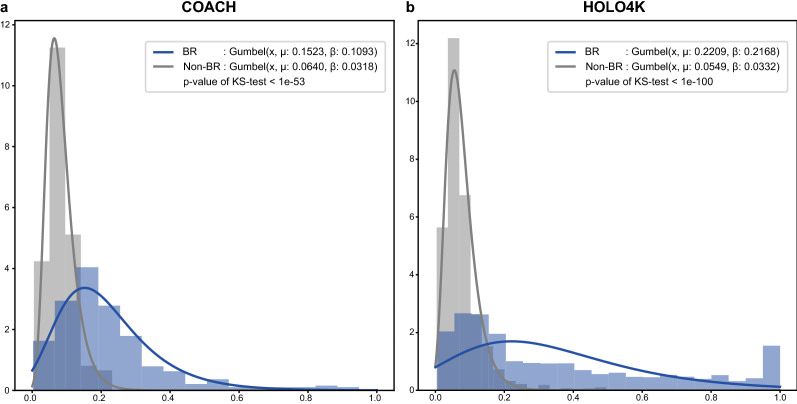
Histograms and Gumbel distributions of maximum attention on compounds for BR/non-BR. We collected the maximum attention values of compound tokens in the first transformer for BR/non-BR from each complex. We fitted the collected maximum attention values to a Gumbel distribution, showing the differences in the distribution of attention on BR and non-BR protein grids. The blue histogram plots the maximum attention values for BR, while the blue line shows the fitted Gumbel distribution. The gray histogram and line analogously represent the non-BR case. **a** Histograms and fitted Gumbel distributions of the COACH dataset. **b** Histograms and fitted Gumbel distributions of the HOLO4K dataset

**Fig. 6:**
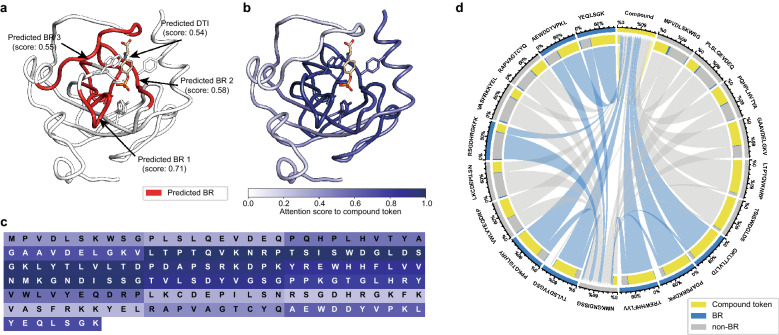
Attention visualizations of DTI pair. We visualized the attention values of protein–ligand pairs to understand the DTI and BR prediction mechanisms for various aspects, such as 3D-complex, sequence, and Circos plot. **a** Predicted DTIs and BRs are mapped to 3D complex 2QYQ, which is in the external test dataset COACH. **b** Attention values to compound token from each protein grid are mapped to 3D complex. Protein grids around the interacting ligand show higher attention values than those in the other regions. **c** Attention values of protein grids are visualized in sequence perspective. **d** Circos plots of attention values were drawn to analyze the interactions between compound tokens and BRs. Each sector represents a compound token and protein grid (BR or non-BR), which are colored as yellow, blue, and gray, respectively. Stroking lines represent their attentions. Source sectors represent queries, sectors represent keys, and line widths represent normalized attention. The Circos plot is of the attention for head 3 of transformer 1 for complex 2QYQ. Protein grids give attention to compound tokens, BRs give the most attention to compound tokens, while non-BRs give to non-BRs rather than compound tokens

As shown above for the BR training datasets, HoTS provided better DTI prediction performance for proteins of the same SCOPe family than CNN-based methods, such as DeepConv-DTI. In HoTS, attention on a compound token in a transformer is designed to reflect the relative importance of protein grids to a given DTI. Accordingly, we inspected the maximum attention value of protein grids for proteins in the COACH and HOLO4K datasets for the transformers, drawing the Gumbel distribution for the first transformers in Fig. 5. In doing so, we noted that attention scores of protein grids for BRs (μ: 0.1523, β: 0.1093 of Gumbel distribution for COACH, μ: 0.2209, β: 0.2168 of Gumbel distribution for HOLO4K) were higher than those for non-BRs (μ: 0.0640, β: 0.0318 of Gumbel distribution for COACH, μ: 0.0549, β: 0.0332 of Gumbel distribution for HOLO4K), thus allowing a compound token to concentrate on grids with higher values that more likely contain BRs, improving DTI prediction performance. In addition, when we conducted a Kolmogorov–Smirnov test to evaluate the difference between BR and non-BR, we achieved extremely low p-values (< 1e−53 for COACH, < 1e−100 for HOLO4K), justifying our hypothesis. To examine a more detailed mechanism of attention, we firstly visualized the complex of *O*-phosphotyrosine and human RAF protein (PDB ID: 2QYQ, [60]), which is predicted as a positive DTI pair in the test dataset, as shown in Fig. 6a. First, for predicting BRs, the attention mechanism in the transformer blocks well-captured the importance of BRs in the given complex (Fig. 6a, b). The top-3 predictions properly indicate binding pocket, and the top-4 prediction is also located at the right helix of the binding pocket with a BR prediction score of 0.52. In addition, from head 3 of transformer 1, we mapped the attention scores to compound token from each protein grid and visualized in 3D structure and sequence, shown in Fig. 6b, c. Also, we can see the attention scores of protein grids that are actual BRs are higher than other protein grids (Figs. 5a, b, 6b). This result indicates that our model correctly predicts DTIs by automatically detecting the BRs as important protein grids. In addition, we drew a Circos plot [45, 46] of heads in the transformers. As depicted in Fig. 6d, we can see that all protein grids give attention to the compound tokens. However, the attention portion of the compound token of BRs is higher than that of non-BRs. BRs give most of the attention to compound tokens, while non-BRs give more attention to the far non-BR grid between BRs (protein grid labeled as “NMKGNDISSG”), modeling the ligand selectivity of BRs. In addition, in head 2 of transformer 2 for the complex of *N*-acetylglucosamine and human Lysosome C (PDB ID: 1LZS [61]), BRs give attention to BRs regardless of their distance, crossing the circle of the Circos plot (Additional file 1: Fig. S5). Compound tokens give high attention to far BRs, and we can see directly how compound tokens aggregate to predict DTIs. In short, by visualizing attention with a Circos plot, we can understand how HoTS transformers play a role in the interdependencies between BRs and aggregating BR information to predict DTIs for compound tokens.

### Docking simulation result based on HoTS prediction results

Because HoTS predicts BRs to the corresponding DTI, HoTS can provide a good starting point for further virtual screening like docking simulation. For a brief example, MARK1 of human does not have any 3D complex with interacting druggable ligand, while it has many interacting compounds tested in binding assays [62, 63]. On the other hand, Staurosporine is known to bind to kinase families [62, 64], and it has a high DTI prediction score of 0.91. Thus, we choose MARK1 and Staurosporine to show an example of the possible usage of HoTS in the virtual screening pipeline. Detailed docking procedures are described in Additional file 1. In preparation for docking simulation, predicted BRs can be mapped to 3D structures, and a search box of docking simulation can be drawn based on predicted BRs (Additional file 2). We confirmed that the search box derived from predicted BRs correctly includes kinase binding motifs (Additional file 1: Fig. S6). Furthermore, we demonstrated the docking result from AutoDock Vina [65] gives high binding energy for the docking of Staurosporine into kinase binding motifs (Additional file 1: Fig. S7, Additional file 2).

### Limitations

Despite the good performance of our model, it can still be improved. First, we may be able to incorporate other compound representations, such as neural fingerprints [66], message passing neural networks [67], and SMILES-BERT [68], which are expected to overcome several limitations when using Morgan/circular fingerprints, to build a fully end-to-end model that is more interpretable. In addition, we may be able to apply image segmentation [69] instead of object detection for BR detection. Although we aimed to predict BRs explicitly for DTIs as a trial study, we will likely be able to predict more delicate motifs by leveraging segmentation techniques. We expect that with greater improvement, our model will be able to predict the exact binding sites and potentially even binding modes.

In addition, HoTS cannot deliver drug action information, such as agonistic/antagonistic, for predicted DTIs. The main reason why HoTS neglects this type of information is that the databases we used in this research do not fully provide enriched drug action knowledge. For example, DrugBank [33] provides limited information like identifiers of drugs, without their detailed molecular mechanism. Therefore, the chemogenomic community should elaborate more efforts to build comprehensive data resources, and we further that better and more interpretable prediction models will be built.

## Conclusions

In this study, we developed a deep learning model called HoTS to predict BRs and DTIs. Firstly, we parsed BRs from 3D complex databases and collected DTIs from various drug databases to train our model to predict BRs and DTI. The model is based on transformers whose inputs are compound token from chemical fingerprints and encoded protein grids representing sequential motifs captured by CNN. HoTS predicts BRs from transformer output of protein grid encoding and DTIs from transformer output of the compound token. As a result, HoTS showed good performance in the BR predictions compared with previous models using 3D-complexes and outperformed the other DTI prediction models. Moreover, we analyzed attention values by quantitative statistical tests and visualized the impacts of attention in diverse aspects. Thus, we can conclude how transformer structures help DTI and BR prediction by emphasizing on BRs and the compound token. Finally, we gave an example that HoTS prediction can provide a good starting point for further drug discovery processes like docking simulation.

## Supplementary Information

**Additional file 1.** Additional information, tables, and figures.

**Additional file 2.** The coordination files of the search box and docking results for MARK1 and Staurosporine.

## Data Availability

All codes are available at https://github.com/GIST-CSBL/HoTS.

## Abbreviations

DTI: Drug–target interaction
PCM: Proteochemometric
CNN: Convolutional neural network
PDB: Protein Data Bank
BI: Binding information
BR: Binding region
IoU: Intersection over union
AP: Average precision
AUPR: Area under the precision–recall curve
