# Supplementary material for: Sequence-based prediction of protein binding regions and drug–target interactions
Source: J Cheminform. 2022 Feb 8;14:5. doi: 10.1186/s13321-022-00584-w (PMC8822694; doi:10.1186/s13321-022-00584-w)
Supplement: Supplementary file 1 — Additional file 1. Additional information, tables, and figures. [file 13321_2022_584_MOESM1_ESM.pdf]

Additional file 1: Sequence-based prediction of protein binding regions and drug–target interactions

Ingoo Lee, Hojung Nam§

School of Electrical Engineering and Computer Science, Gwangju Institute of Science and Technology (GIST), Buk-gu, Gwangju 61005, Republic of Korea.

§ Corresponding author

E-mail addresses:

IL: dlsrnsladlek@gist.ac.kr

HN: hjnam@gist.ac.kr

Table of contents

Dataset generation and statistics 3

3D complexes and binding region generation 3

Model details and training scheme 5

Model details 5

Transformers 5

Attention analysis 7

Training scheme 8

Binding region prediction results 11

DTI prediction performances comparison (Benchmark dataset in TransformerCPI) 12

Additional Circos plot 13

Docking simulation based on HoTS prediction results 14

Docking methods 14

Docking results 15

References 17

Dataset generation and statistics

3D complexes and binding region generation

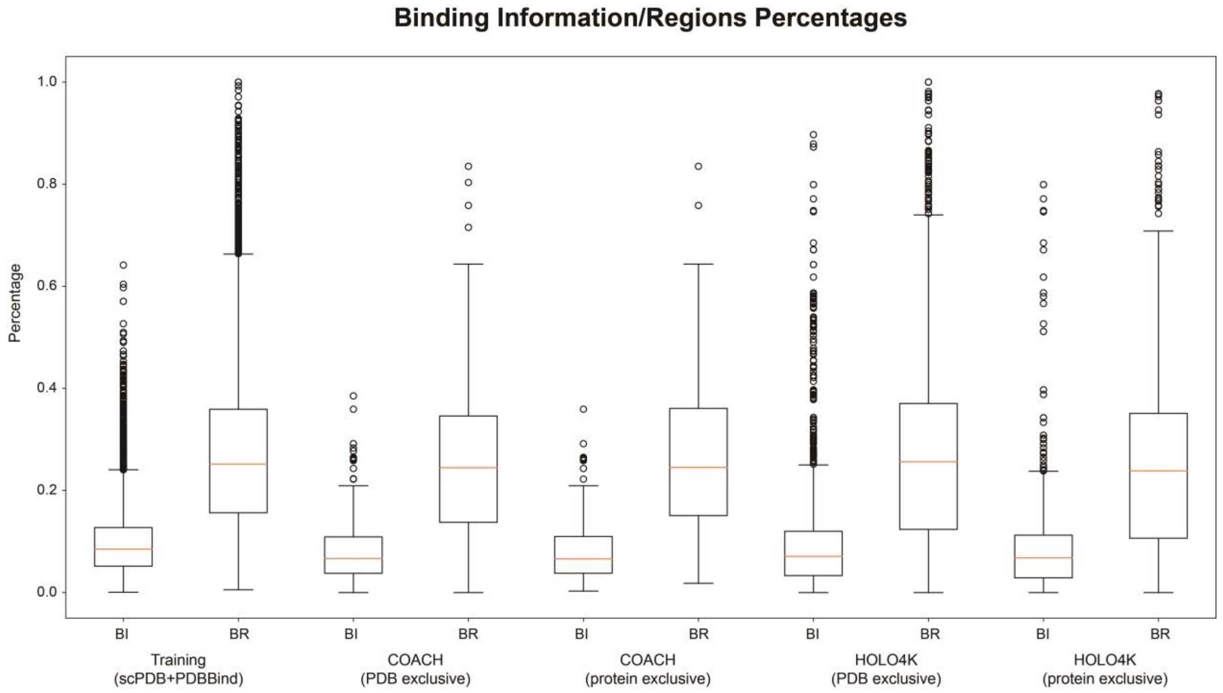

Fig. S1. Percentages of amino acids at binding sites and BRs

We inspected the percentages of amino acids at binding sites for the collected datasets. The median percentages were approximately 7–9% for all datasets. After converting binding sites to BRs, the percentages of amino acids at BRs increased approximately three-fold. Although all BI expanded, the percentages of amino acids in the merged BRs did not increase arithmetically, indicating that the binding sites were clustered at interacting motifs.

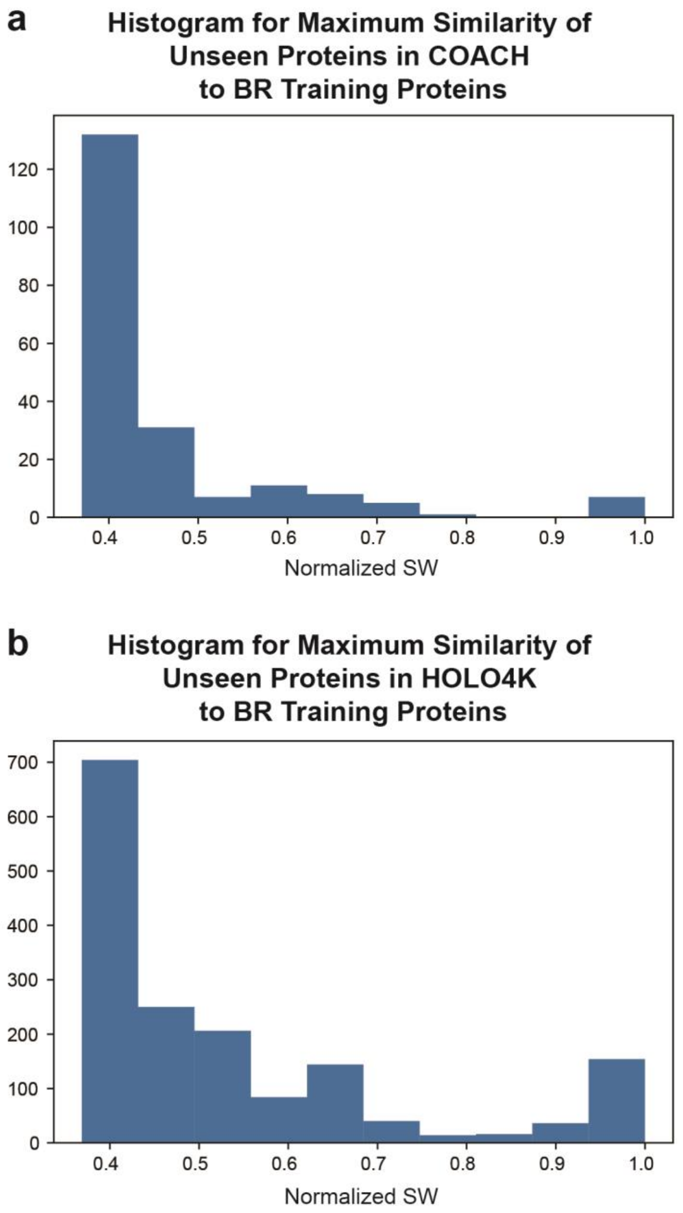

Fig. S2. Maximum sequence similarities between BR training proteins and BR test proteins.

We calculated the sequence-normalized Smith–Waterman score as the similarity between BR training proteins and BR test proteins. Histograms of the maximum similarity of BR test proteins are plotted for the **a**. COACH dataset and **b**. HOLO4K dataset.

Model details and training scheme

Model details

HoTS comprises two models: BR prediction and DTI prediction models. The BR prediction model predicts the locations, lengths, and confidence scores of the BRs for whole protein sequences and their ligands. Meanwhile, the DTI prediction model predicts DTIs from a compound–protein pair. The two models share core layers, including convolution layers, to extract interacting motifs, lower transformers, and fully connected layers to featurize the chemical fingerprints of compounds.

Transformers

The transformers [1] in HoTS are core modules that determine the interactions between compounds and proteins and their interdependencies. The BR prediction model models the interdependencies between protein grid encodings and their ligand selectivity. In the DTI prediction model, more transformers are stacked on the BR prediction model. Because transformers model both the interdependency between protein grid encodings and the interactions between compounds and proteins, we can utilize them as "aggregating" modules to predict DTIs. At the last transformer in the DTI prediction model, HoTS predicts the DTIs from a compound token.

A transformer has several components, including positional encoding and multi-head attention, which help the model to understand positional information and interdependencies, respectively.

Positional encoding

Because HoTS does not use a recurrent neural network, it is unable to encode positional information by the model itself. To encode positional information, we built a positional

encoding (PE) layer and added it to the protein grid encodings. Positional encoding is generated by the following formula:

$$\mathrm{PE}_{(\mathrm{pos},2\mathrm{i})} = \sin\left(pos/10000^{\frac{2i}{d_{model}}}\right)$$
$$\mathrm{PE}_{(\mathrm{pos},2\mathrm{i}+1)} = \cos\left(pos/10000^{\frac{2i}{d_{model}}}\right) \tag{1}$$

where pos is an actual index in the sequence and i is an index of the dimension in the embedding. For low dimensions, PE values differ with position in the sequence, whereas PE values in high dimensions do not. As a result, adding PE affects the inner product, which has a larger value for closer words.

Multi-head attention

Interdependency in a protein sequence is important for determining the 3D shape, function, and interaction with compounds. We utilize self-attention modules to determine interdependencies in proteins and DTIs. In the transformer mechanism, the inputs, i.e., sequences of words, are transformed into queries, keys, and values. The similarities between queries and keys are calculated from their inner products. The similarities between the queries and keys are normalized by the SoftMax function, and the values are then summed by the normalized similarities of the corresponding keys:

$$\mathrm{V}^{\mathrm{n}+1} = \sum_{\mathrm{i}} \mathrm{Softmax}\left(\frac{\mathrm{QK}_{\mathrm{i}}^{\mathrm{T}}}{\sqrt{d_k}}\right) V^n \tag{2}$$

where Q denotes queries, K denotes keys, V denotes values, and $\mathrm{d_k}$ denotes the dimensions of Q and K.

As an elaboration of the attention mechanism, we apply multi-head attention, which considers many aspects of attention with smaller parameters. In multi-head attention, the dimensions of queries, keys, and values are divided into N sub-dimensions. Attention from

the sub-dimensions of queries, keys, and values is then calculated. The resulting values are concatenated, giving the same dimensions as the original queries, keys, and values:

$$\mathrm{MultiHead}(\mathrm{Q},\mathrm{K},\mathrm{V}) = \mathrm{Concat}(head_1, \ldots, head_N)W^O$$
$$\text{where } \mathrm{head_i} = \mathrm{Attention}(QW_i^Q, KW_i^K, VW_i^V) \tag{3}$$

where W indicates the linear transformation of queries, keys, and values. The original values are added to the weighted sum of the values in a residual manner and normalized with layer normalization. In HoTS, multi-head attention modules use grid encodings as inputs to determine interdependencies.

Attention analysis

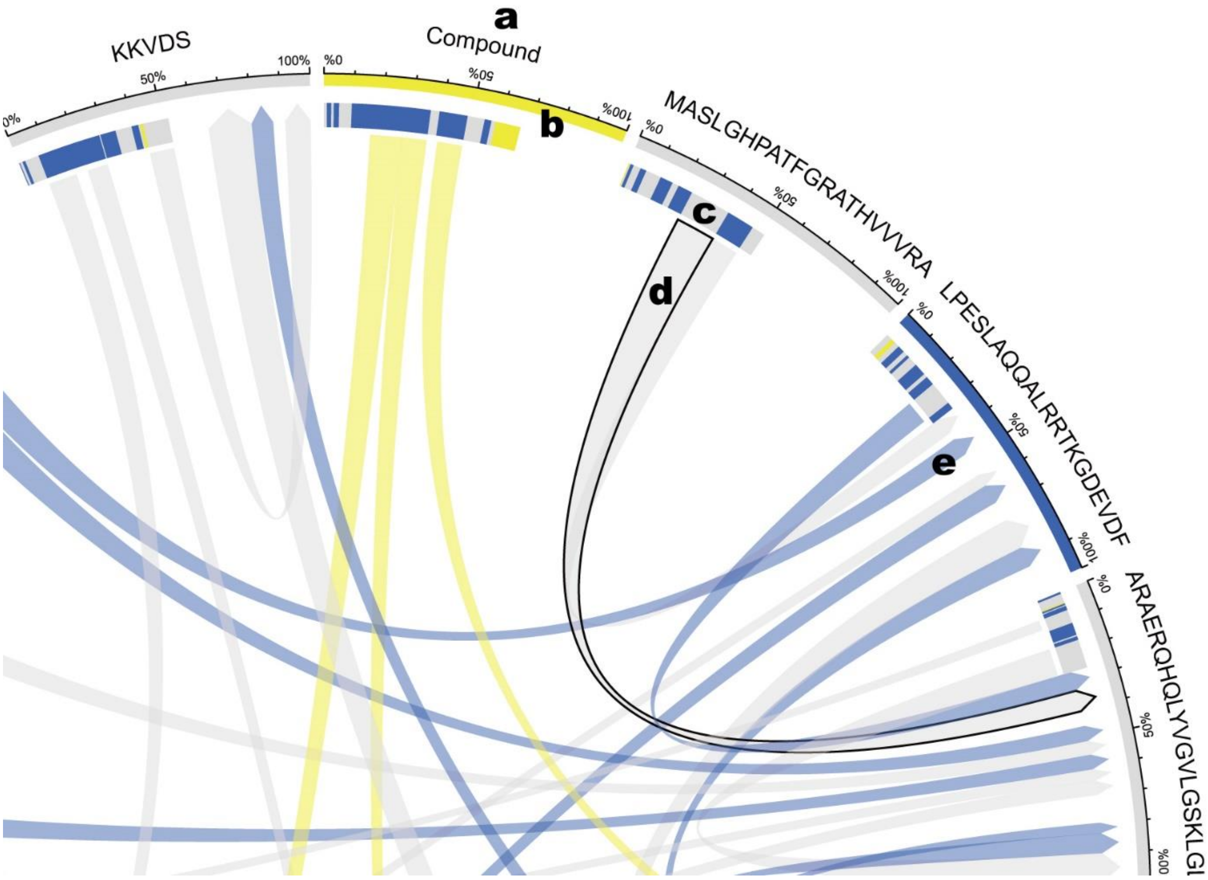

Fig. S3. Explanation of Circos plot.

We drew Circos plots [2] with attention on the head in a transformer using the “circlize” library [3] in R. For better visualization, we scaled sectors corresponding to the compound tokens and protein grids. Each sector starts from a compound token and ends on a protein grid, moving clock-wise. **a**. Label of sector, either a compound token or protein grid. **b**. Sector colored by type. Compound tokens, BRs, and non-BRs are depicted in yellow, blue, and gray, respectively. **c**. Attention given to sectors as a query, the width of which reflects the attention value (colored by target [key] type). **d**. Stroking line to the target (key), whose color corresponds to the source (query) sector. We denote the attention between a compound token as a query and last protein grid. “MASLGHPATFGRATHVVVRA” was used as the query and “ARAERQHQLYVGVLGSKLGL” as key. Only the attention values in the upper 90th percentile are visible. e) Attention received from other sectors as a key, depicted as a large arrow. Only the attention values in the upper 90th percentile are visible.

Training scheme

Before modeling the interdependencies via transformers, protein grid encodings are featured by a convolutional neural network (CNN). During training of the BR and DTI prediction models, we noted that the CNN layers did not converge on different tasks. To solve this problem, we set half of the CNN layers to be trained during the training of the BR prediction model and the other half during the training of the DTI prediction model. Other parameters are dominated by the DTI prediction model, which does not decrease the BR prediction performance.

After finishing the pretraining, we alternated between training the BR prediction model and the DTI prediction model. As indicated in **Fig. S4**, BR prediction performance converged quickly. The selected hyperparameters are listed in **Table S1**.

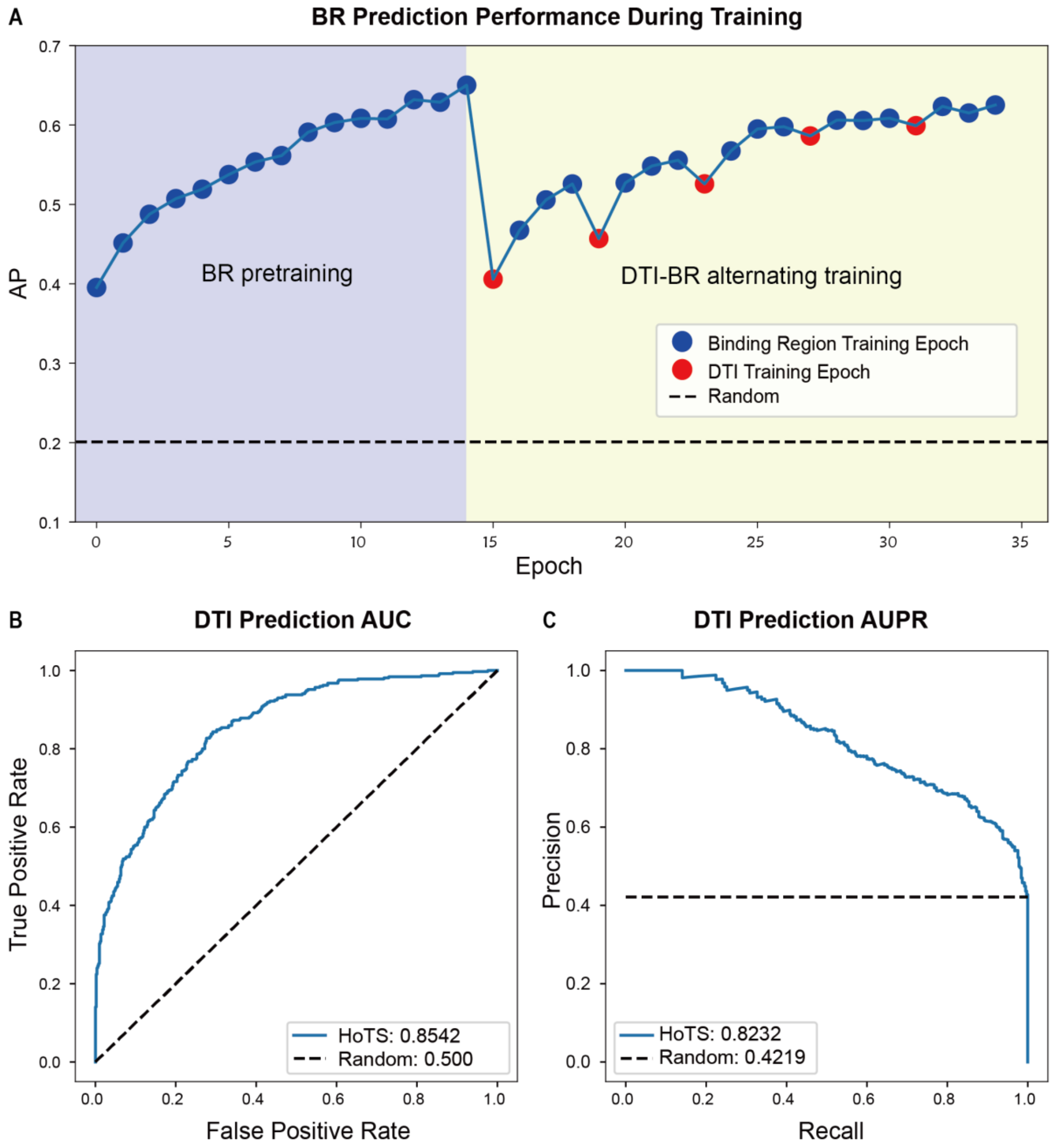

Fig. S4. Validation of the performances of the HoTS models.

a. Performance on the BR training datasets. Blue background represents BR pretraining epochs and yellow background represents DTI–BR alternating training epochs. **b**. Area under the receiver operating curve for DTI prediction. **c**. AUPR for DTI prediction.

Table S1 - Selected hyperparameter values for deep learning model

Hyperparameter	Selected value

Learning rate	0.0001
Decay rate	0.0001
Dropout ratio	0.1
Spatial 1D dropout ratio on embedding layer	0.2
Threshold	0.35
Regression loss weight	0.1
Negative loss weight	0.1
BR prediction anchor	[9]
Focal loss weight	0.2
Protein grid length	10
Pretraining epochs	15
DTI training epochs	5
BR training per DTI training	3
Activation function	GELU
Amino acid embedding dimensions	20
No. of filters	128
Protein CNN windows	5, 10, 15, 20, 25, 30
Hidden dimensions of transformers	128
No. of heads in multi-head attention	4
No. of transformers for BR	2
No. of transformers for DTI	4
Fully connected layers for the protein convolution layers	128
Fully connected layers for drug tokens	512,128
Fully connected layers for BR prediction	256, 64

Fully connected layers after concatenating layers for DTI prediction	256, 64

Abbreviations: CNN, convolutional neural network; BR, binding region; DTI, drug–target interaction

Binding region prediction results

Table S2 – Performance of COACH and HOLO4K BR prediction with different sequence similarity thresholds

	Threshold	0.9	0.8	0.7	0.6	0.5
COACH	No. of complexes	126	126	123	114	103
	No. of proteins	126	126	123	114	103
	Top-n success rate	61.9±1.5%	61.9±1.5%	61.6±1.5%	60.9±2.0%	60.8±1.8%
	Top-(n+2) success rate	82.4±0.9%	82.4±0.9%	82.6±0.9%	81.6±1.1%	81.7±0.9%
HOLO 4K	No. of complexes	619	604	589	527	436
	No. of proteins	251	368	357	319	251
	Top-n success rate	53.1±0.8%	52.6±0.7%	52.3±0.8%	49.7±0.9%	47.6±1.ㄴ 5%
	Top-(n+2) success rate	71.5±0.5%	71.2±0.5%	71.0±0.7%	69.0±0.8%	66.5±0.8%

Similarity metric is normalized Smith–Waterman score

DTI prediction performances comparison (Benchmark dataset in TransformerCPI)

Table S3 – DTI prediction performance on the BindingDB dataset [4]. The performance scores of the previous models are directly from the TransformerCPI paper [5].

Method	AUC	Precision
GraphDTA	0.929	0.917
GCN	0.927	0.913
CPI–GNN	0.603	0.543
TransformerCPI	0.951	0.949
HoTS	**0.966**	**0.965**

Table S4 – DTI prediction performance on the Human dataset [6]. The performance scores of the previous models are directly from the TransformerCPI paper [5].

Method	AUC	Precision	Recall
KNN	0.860	0.927	0.798
RF	0.940	0.897	0.861
L2	0.911	0.913	0.867
SVM	0.910	**0.966**	**0.969**
GraphDTA	0.960 ± 0.005	0.882 ± 0.040	0.912 ± 0.040
GCN	0.956 ± 0.004	0.862 ± 0.006	0.928 ± 0.010
CPI–GNN	0.970	0.918	0.923
DrugVQA (VQA-seq)	0.964 ± 0.005	0.897 ± 0.004	0.948 ± 0.003
TransformerCPI	0.973 ± 0.002	0.916 ± 0.006	0.925 ± 0.006
HoTS (Proposed)	**0.985 ± 0.001**	0.926 ± 0.003	0.952 ± 0.004

Table S5 – DTI prediction performance on the *C. elegans* dataset [6]. The performance scores of the previous models are directly from the TransformerCPI paper [5].

Method	AUC	Precision	Recall
KNN	0.858	0.801	0.827
RF	0.902	0.821	0.844
L2	0.892	0.890	0.877
SVM	0.894	0.785	0.818
GraphDTA	0.974 ± 0.004	0.927 ± 0.015	0.912 ± 0.023
GCN	0.975 ± 0.004	0.921 ± 0.008	0.927 ± 0.006
CPI–GNN	0.978	0.938	0.929
TransformerCPI	0.988 ± 0.002	0.952 ± 0.006	**0.953 ± 0.005**
HoTS (Proposed)	**0.988 ± 0.001**	**0.954 ± 0.003**	0.949 ± 0.003

Additional Circos plot

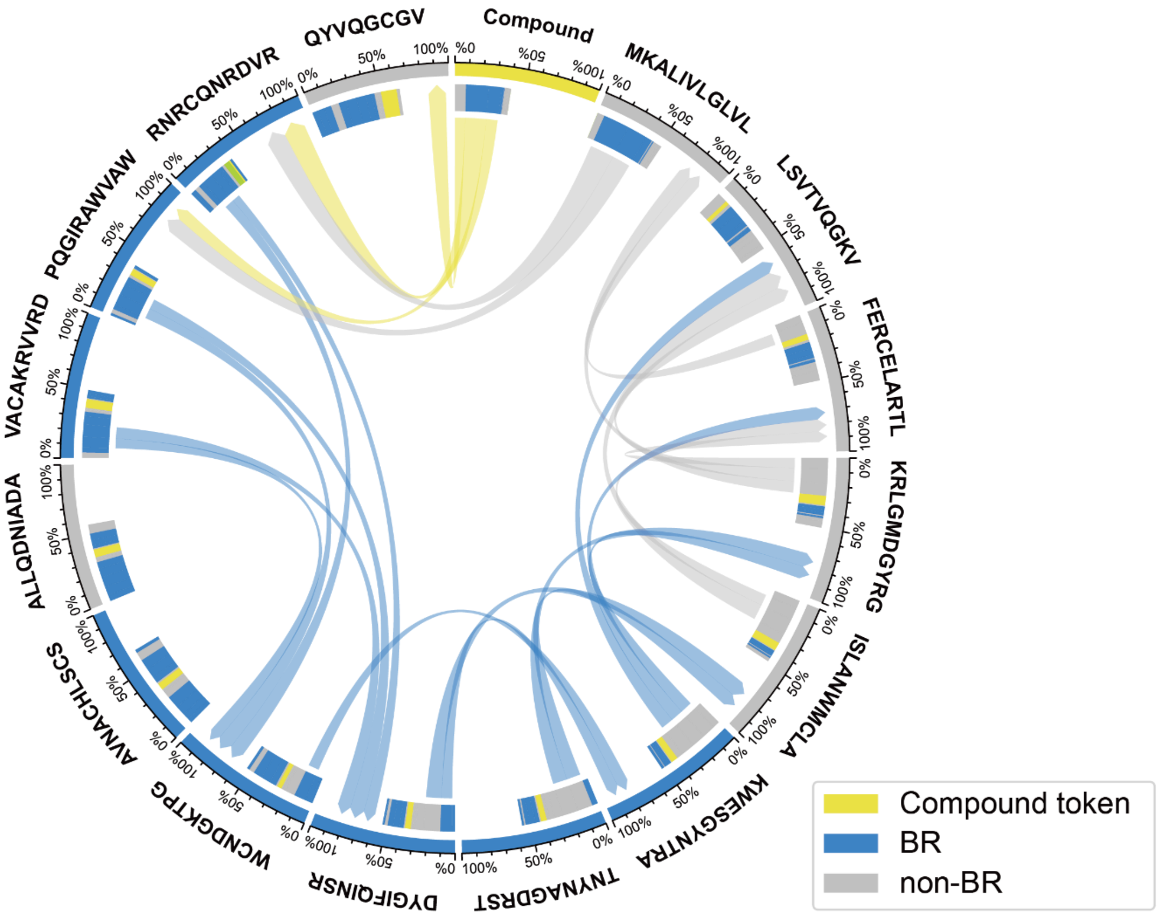

Fig. S5. Circos plot of 1LZS.

Circos plot of attention for head 2 of transformer 2 for complex 1LZS [7]. Compound tokens give attention to BRs rather than non-BRs, as shown by the yellow stroking lines and their targeting colors. In addition, BRs give attention to each other, crossing the Circos circle.

s

Docking simulation based on HoTS prediction results

Docking methods

From the predicted binding regions and DTIs, HoTS could provide a good starting point for the further virtual screening process. To demonstrate an example based on HoTS predictions, we selected the crystal structure of serine/threonine-protein kinase MARK1, whose protein is not in the HoTS training dataset but is homologous to it (Uniprot ID: Q9P0L2, PDB ID: 2HAK [8]). Moreover, MARK1 does not have any complex structures, consequently, we do not know its binding information. Because part of the residues are missing in PDB, we recovered the missing residues by using PDBFixer [9]. We selected Staurosporine (PubChem Compound ID: 44259), which is already known to bind many kinases [10, 11], as the ligand to simulate. Staurosporine gives a DTI prediction score of 0.91 from HoTS and is known to have a high binding affinity to MARK1 in binding assay [12, 13]. The 3D structure of the ligand was downloaded from PubChem. The ligand and receptor were prepared by using MGLTools [14]. For running AutoDock Vina [15], the search space was drawn from the BRs predicted from HoTS. The other parameters for AutoDock Vina were set to default. The search space and result complex were visualized via UCSF Chimera [16].

Docking results

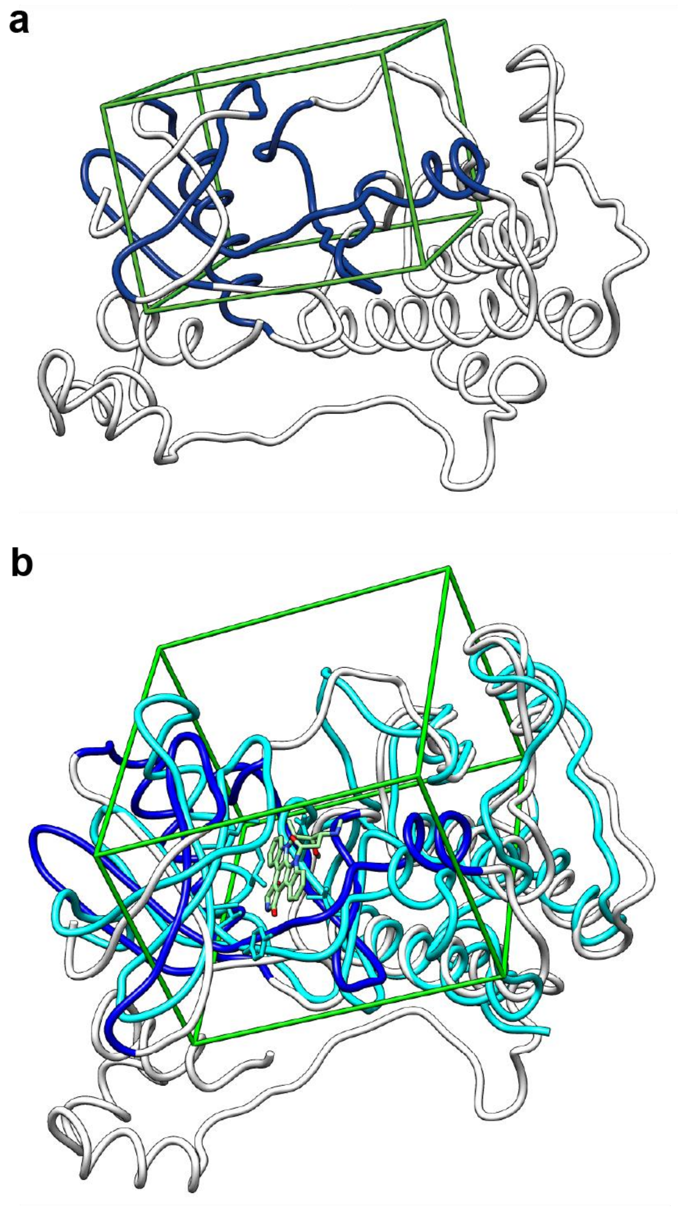

Fig. S6. Structural alignment results for 2HAK and 2DQ7 and search space.

HoTS can provide a good starting point for further virtual screening. We selected the 3D structure of MARK1 (PDB ID: 2HAK [8]) and Staurosporine as an example. The search space was drawn based on HoTS prediction and compared with the homologous complex **a**. Predicted BRs of MARK1–Staurosporine pair are mapped on the 3D structure, and the search space of docking simulation is drawn based on BR predictions. **b**. Tyrosine-protein kinase Fyn has a 3D complex with Staurosporine (PDB ID: 2DQ7, [17]). We executed structural

alignment between Fyn with Staurosporine and MARK1 (PDB ID: 2HAK), with the search space drawn from the predicted BRs. As can be seen, the structures of Fyn and MARK1 are structurally aligned with a root mean square deviation of 1.191 Å. The relocated Staurosporine is included in the search space drawn by the predicted BRs from the MARK1–Staurosporine pair. Corresponding coordination file is provided in Additional file 2.

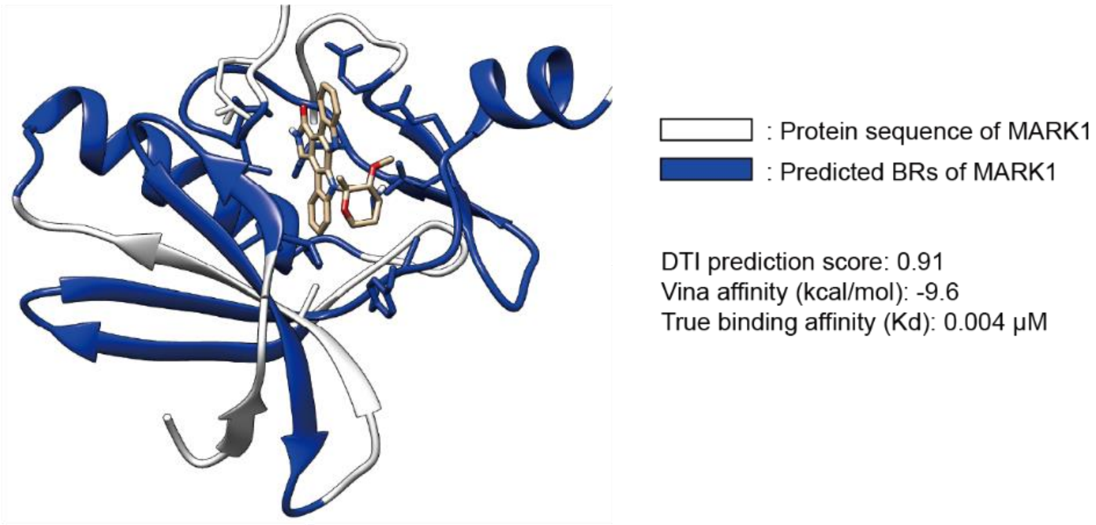

Fig. S7. Docking results of MARK1 and Staurosporine

Docking results of MARK1 and Staurosporine obtained by using AutoDock Vina [15]. Corresponding coordination file is provided in Additional file 2.

References

1. Vaswani A, Shazeer N, Parmar N, Uszkoreit J, Jones L, Gomez AN, et al. (2017) Attention is all you need. arXiv:1706.03762.
2. Krzywinski M, Schein J, Birol I, Connors J, Gascoyne R, Horsman D, et al. (2009) Circos: an information aesthetic for comparative genomics. Genome Res 19(9):1639-1645. doi:10.1101/gr.092759.109.
3. Gu Z, Gu L, Eils R, Schlesner M, Brors B (2014) circlize implements and enhances circular visualization in R. Bioinform 30(19):2811-2812. doi:10.1093/bioinformatics/btu393.
4. Gao KY, Fokoue A, Luo H, Iyengar A, Dey S, Zhang P (2018) Interpretable drug target prediction using deep neural representation. Twenty-Seventh International Joint Conference on Artificial Intelligence, 2018: 3371-3377.
5. Chen L, Tan X, Wang D, Zhong F, Liu X, Yang T, et al. (2020) TransformerCPI: improving compound-protein interaction prediction by sequence-based deep learning with self-attention mechanism and label reversal experiments. Bioinform 36(16):4406-4414. doi:10.1093/bioinformatics/btaa524.
6. Tsubaki M, Tomii K, Sese J (2019) Compound-protein interaction prediction with end-to-end learning of neural networks for graphs and sequences. Bioinform 35(2):309-318. doi:10.1093/bioinformatics/bty535.
7. Song H, Inaka K, Maenaka K, Matsushima M (1994) Structural changes of active site cleft and different saccharide binding modes in human lysozyme co-crystallized with hexa-N-acetyl-chitohexaose at pH 4.0. J Mol Biol 244(5):522-540. doi:10.1006/jmbi.1994.1750.
8. Marx A, Nugoor C, Muller J, Panneerselvam S, Timm T, Bilang M, et al. (2006) Structural variations in the catalytic and ubiquitin-associated domains of microtubule-

associated protein/microtubule affinity regulating kinase (MARK) 1 and MARK2. J Biol Chem 281(37):27586-27599. doi:10.1074/jbc.M604865200.

9. Eastman P, Friedrichs MS, Chodera JD, Radmer RJ, Bruns CM, Ku JP, et al. (2013) OpenMM 4: A reusable, extensible, hardware independent library for high performance molecular simulation. J Chem Theory Comput 9(1):461-469. doi:10.1021/ct300857j.

10. Fabian MA, Biggs WH, 3rd, Treiber DK, Atteridge CE, Azimioara MD, Benedetti MG, et al. (2005) A small molecule-kinase interaction map for clinical kinase inhibitors. Nat Biotechnol 23(3):329-336. doi:10.1038/nbt1068.

11. Davis MI, Hunt JP, Herrgard S, Ciceri P, Wodicka LM, Pallares G, et al. (2011) Comprehensive analysis of kinase inhibitor selectivity. Nat Biotechnol 29(11):1046-1051. doi:10.1038/nbt.1990.

12. Karaman MW, Herrgard S, Treiber DK, Gallant P, Atteridge CE, Campbell BT, et al. (2008) A quantitative analysis of kinase inhibitor selectivity. Nat Biotechnol 26(1):127-132. doi:10.1038/nbt1358.

13. Narayan S, Ramisetti S, Jaiswal AS, Law BK, Singh-Pillay A, Singh P, et al. (2019) ASR352, a potent anticancer agent: synthesis, preliminary SAR, and biological activities against colorectal cancer bulk, 5-fluorouracil/oxaliplatin resistant and stem cells. Eur J Med Chem 161:456-467. doi:10.1016/j.ejmech.2018.10.052.

14. Morris GM, Huey R, Lindstrom W, Sanner MF, Belew RK, Goodsell DS, et al. (2009) AutoDock4 and AutoDockTools4: automated docking with selective receptor flexibility. J Comput Chem 30(16):2785-2791. doi:10.1002/jcc.21256.

15. Trott O, Olson AJ (2010) AutoDock Vina: improving the speed and accuracy of docking with a new scoring function, efficient optimization, and multithreading. J Comput Chem 31(2):455-461. doi:10.1002/jcc.21334.

16. Pettersen EF, Goddard TD, Huang CC, Couch GS, Greenblatt DM, Meng EC, et al. (2004) UCSF Chimera--a visualization system for exploratory research and analysis. J Comput Chem 25(13):1605-1612. doi:10.1002/jcc.20084.

17. Kinoshita T, Matsubara M, Ishiguro H, Okita K, Tada T (2006) Structure of human Fyn kinase domain complexed with staurosporine. Biochem Biophys Res Commun 346(3):840-844. doi:10.1016/j.bbrc.2006.05.212.
